# Collagenous gastritis in pediatric patients: Two case reports and literature review

**DOI:** 10.1097/MD.0000000000050709

**Published:** 2026-09-18

**Authors:** Baoqi Chen, Haiyan Liu, Jinming Gong, Yuan Chen, Wenxin Shi, Lu Guo, Lifeng Sun

**Affiliations:** a Department of Pediatrics, Provincial Hospital Affiliated to Shandong First Medical University, Jinan, Shandong, China.

**Keywords:** Anemia, Collagenous gastritis, Masson’s trichrome staining, Pediatric

## Abstract

**Rationale:**

Collagenous gastritis (CG) is a rare disorder of unclear etiology. We report two pediatric cases and provide a comprehensive review of the literature.

**Patient concerns:**

Case 1 was a 7-year-6-month-old girl presenting with severe iron-deficiency anemia and pallor. Esophagogastroduodenoscopy (EGD) revealed fragile, bleeding-prone gastric mucosa with diffuse inflammatory changes. Histopathological examination of gastric biopsy specimens demonstrated subepithelial collagen deposition with positive Masson’s trichrome staining, confirming the diagnosis of CG. Case 2 was a 12-year-1-month-old girl presenting with recurrent anemia and fatigue. EGD showed thickened gastric folds and pale mucosa. Gastric biopsy demonstrated collagen-like fiber deposition beneath the surface epithelium with positive Masson’s trichrome staining.

**Diagnoses:**

Both patients were diagnosed with CG based on characteristic histopathological findings, including subepithelial collagen deposition and positive Masson’s trichrome staining.

**Interventions:**

Both patients received supportive treatment, including gastric mucosal protection and iron supplementation for anemia.

**Outcomes:**

During follow-up, both patients showed improvement in anemia without disease progression. No recurrence of anemia was observed in Case 1 during one year of follow-up. In Case 2, anemia-related symptoms improved after treatment, and the patient remained clinically stable at 8 months of follow-up.

**Lessons:**

Pediatric CG commonly presents with abdominal pain and iron-deficiency anemia, whereas endoscopic findings may vary among different gastric regions. Histopathological confirmation showing subepithelial collagen bands with positive Masson’s trichrome staining is essential for diagnosis. CG should be considered in children with unexplained iron-deficiency anemia, especially when accompanied by gastrointestinal symptoms. Early recognition and appropriate gastric biopsy during EGD may help prevent delayed diagnosis.

## 1. Introduction

Collagenous gastritis (CG) is a rare gastric disorder first described by Colletti et al in 1989.^[1]^ The disease exhibits distinct clinical characteristics between pediatric and adult patients. Pediatric patients commonly present with abdominal pain and iron-deficiency anemia, whereas adult patients more frequently exhibit extensive gastrointestinal involvement, particularly when associated with other collagenous gastrointestinal disorders, such as collagenous colitis, which may manifest as chronic watery diarrhea. Histologically, CG is defined by abnormal thickening of the subepithelial collagen band in the gastric mucosa (≥10μm), accompanied by chronic inflammatory cell infiltration. The pathogenesis remains unclear, and current treatment strategies are primarily based on symptomatic and supportive management. Due to its rarity, CG is often underrecognized in clinical practice and may be misdiagnosed or diagnosed after a prolonged delay. Therefore, increasing awareness of its clinical and pathological features is important for early diagnosis and appropriate management.^[1]^ In this study, we describe two pediatric cases of CG and review previously reported cases to summarize the clinical manifestations, endoscopic features, histopathological findings, treatment approaches, and follow-up outcomes. Our findings aim to improve recognition of CG among pediatric clinicians and facilitate early diagnosis.

## 2. Case data

### 2.1. Case 1

**Figure 1.**
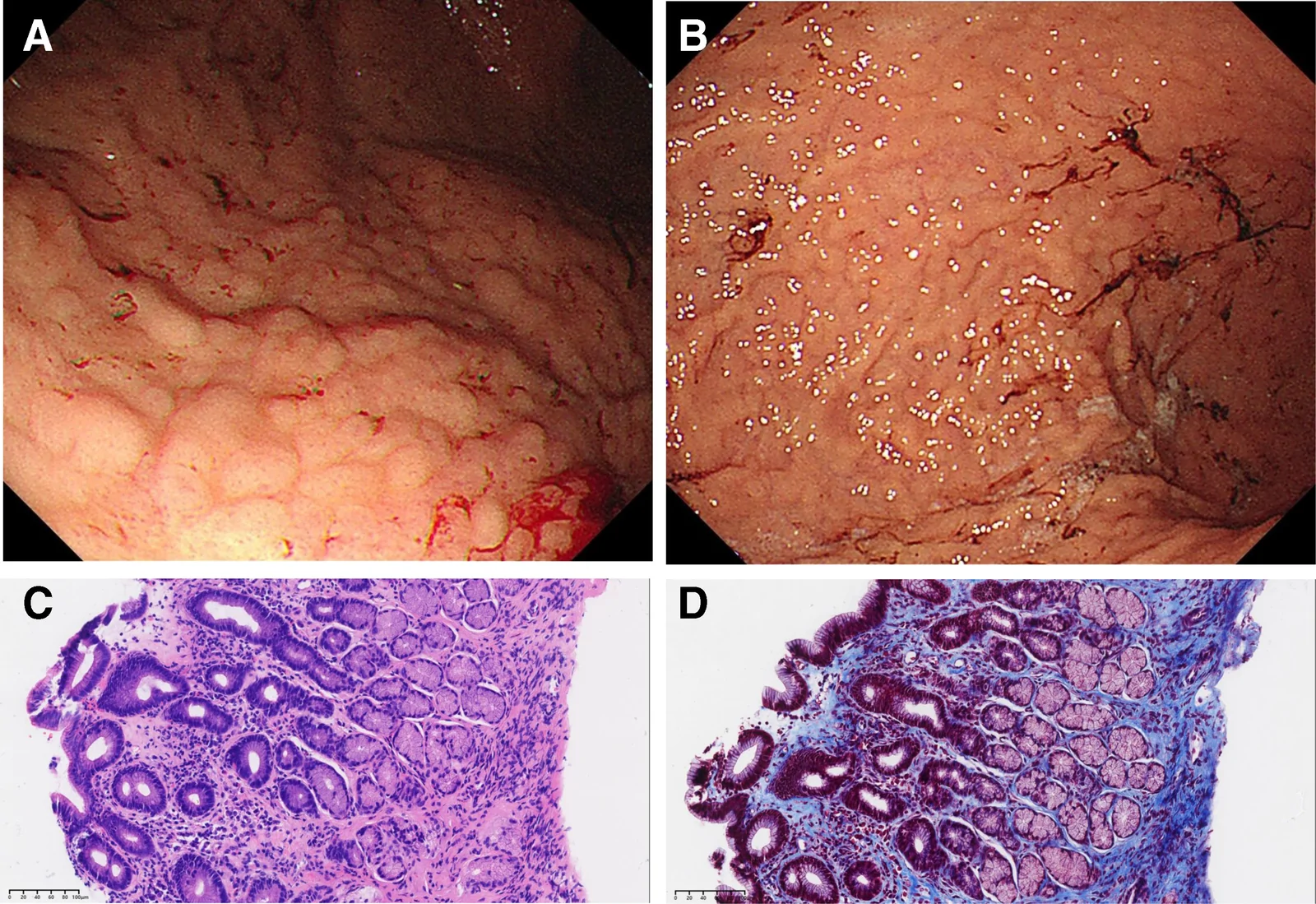
Endoscopic and histopathological findings in Case 1. (A, B) Endoscopic images showing rough and edematous gastric body mucosa with diffuse granular changes and mucosal friability. (C, D) Histopathological examination of gastric biopsy specimens stained with hematoxylin and eosin (H&E) (left, ×200) and Masson’s trichrome staining (right, ×200). H&E staining demonstrates a markedly thickened subepithelial collagen band composed of homogeneous eosinophilic material beneath the surface epithelium, accompanied by inflammatory cell infiltration in the lamina propria. Regenerative changes of the gastric foveolar epithelium and architectural abnormalities of the gastric glands are also observed. Masson’s trichrome staining highlights irregular and markedly thickened collagen deposition beneath the surface epithelium.

A 7-year-6-month-old girl was admitted to our hospital because of persistent pallor lasting for more than one month. At a local hospital, laboratory examination revealed severe anemia, with the lowest hemoglobin (Hb) level of 39 g/L. Despite multiple transfusions of washed packed red blood cells, her Hb level increased only transiently to 80 g/L. EGD performed at the local hospital suggested chronic non-atrophic gastritis; however, CG was not diagnosed at that time. Before admission, she received only oral iron supplementation as symptomatic treatment. Physical examination revealed an anemic appearance. Cardiopulmonary examination showed no abnormalities. Abdominal examination revealed a soft abdomen with tenderness in the epigastric and periumbilical regions. No hepatosplenomegaly was detected. Her past medical history, personal history, and family history were unremarkable. Laboratory examination after admission showed an Hb level of 89 g/L and a hematocrit level of 29.70%. Red blood cell indices indicated microcytic hypochromic anemia, including a mean corpuscular volume (MCV) of 73.50 fL, mean corpuscular hemoglobin (MCH) of 22.10 pg, and mean corpuscular hemoglobin concentration (MCHC) of 300 g/L. Other routine laboratory parameters were within normal limits.Iron metabolism evaluation showed elevated erythropoietin (55.90 mIU/mL) and soluble transferrin receptor levels (57.21 nmol/L), reduced serum iron (1.17 μmol/L), increased unsaturated iron-binding capacity (68.89 μmol/L), decreased transferrin saturation (1.67%), and low ferritin levels (10.96 ng/mL), suggesting iron deficiency. The ^13^C urea breath test was negative. Urinalysis and stool examination both demonstrated weakly positive occult blood, suggesting possible gastrointestinal blood loss. Further gastrointestinal evaluation was therefore performed. Computed tomography enterography showed localized thickening of the ileal wall; however, no definitive evidence of inflammatory bowel disease or collagenous enteritis was identified. Capsule endoscopy revealed residual old blood in the jejunum, a suspected mucosal ulcer covered with whitish exudate, and another area of mucosal erosion. The intestinal contents appeared dark, and clustered lymphoid follicles were observed in the terminal ileum. No abnormalities were identified in the remaining intestinal mucosa. One month after the initial EGD at the local hospital, repeat EGD was performed at our institution. The gastric body mucosa appeared rough and edematous, with diffuse granular elevations. The mucosa was fragile and prone to bleeding. The gastric fundus mucosa appeared smooth, with a small amount of old blood deposited in the mucus lake (Fig. 1A, B). The rapid urease test was negative. Histopathological examination of gastric biopsy specimens demonstrated severe chronic inflammation with focal lymphocytic infiltration in both the gastric antrum and body. Fibrous tissue deposition was observed in the lamina propria, with focal collagen deposition beneath the surface epithelium.In the gastric body mucosa, a subepithelial collagen band was identified beneath the surface epithelium by special staining. Masson’s trichrome staining highlighted the subepithelial collagen deposition(Fig. 1 C, D). Colonoscopy revealed no significant abnormalities.

After completion of EGD, the patient received hemostatic therapy, gastric mucosal protection therapy, and oral iron supplementation. After discharge, she continued treatment with oral aluminum–magnesium carbonate chewable tablets, omeprazole, L-glutamine sodium oligopeptide granules, and polysaccharide–iron complex. During 6 months of follow-up, the patient did not experience recurrent anemia, although occasional abdominal pain occurred. During one year of follow-up, she received omeprazole treatment for approximately 6 months in total. Repeated complete blood counts showed Hb levels consistently above 100 g/L, and she reported no further abdominal pain. Repeat EGD was not performed because the family declined further endoscopic evaluation.

### 2.2. Case 2

**Figure 2.**
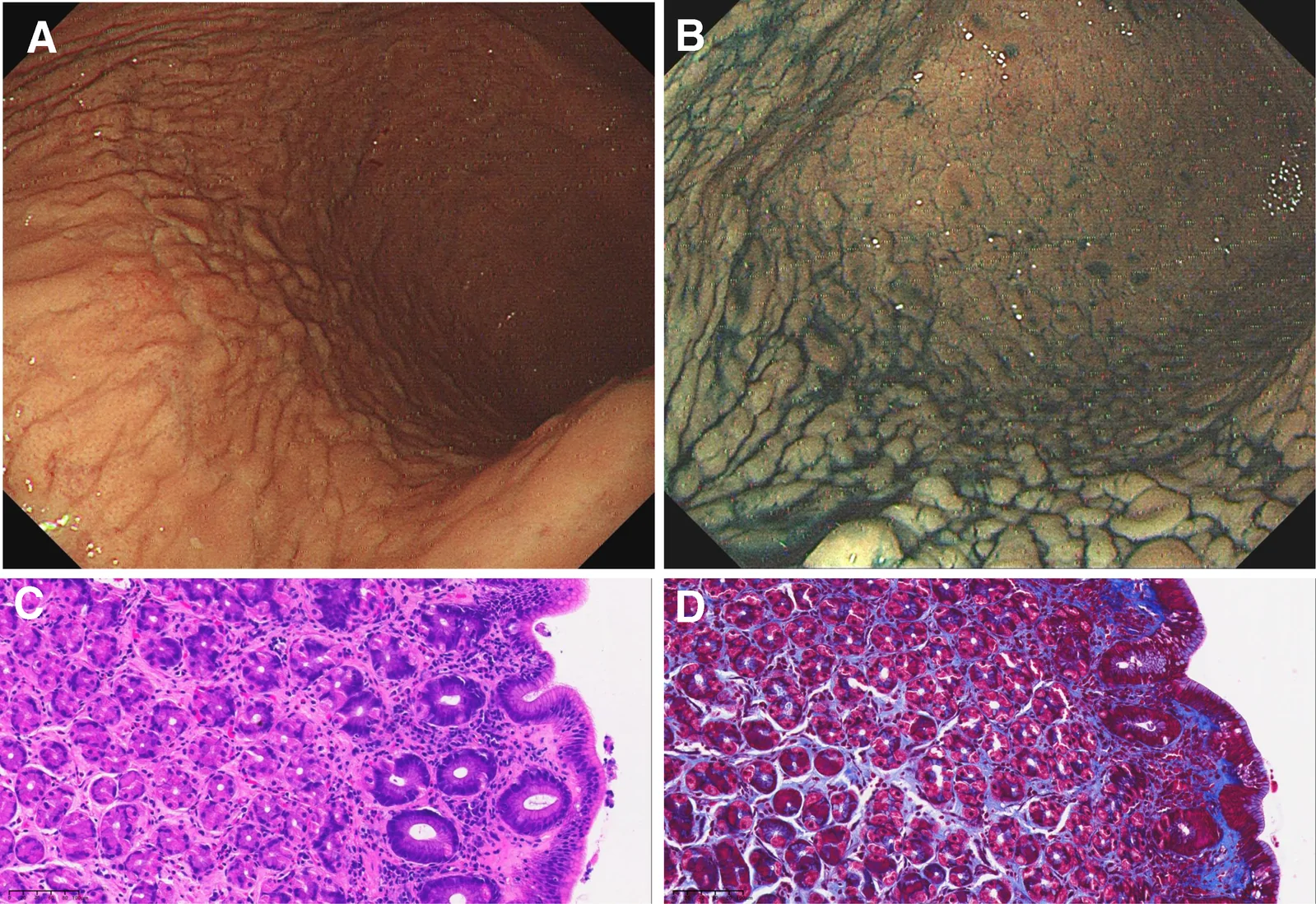
Endoscopic and histopathological findings in Case 2. (A, B) Endoscopic images showing thickened gastric folds with pale mucosa in the gastric body and fundus. (C, D) Histopathological examination of gastric biopsy specimens stained with H&E (left, ×200) and Masson’s trichrome staining (right, ×200). H&E staining demonstrates homogeneous eosinophilic material deposition beneath the surface epithelium within the lamina propria, accompanied by inflammatory cell infiltration. Masson’s trichrome staining highlights diffuse collagen deposition in the lamina propria, presenting as band-like or patchy distributions.

A 12-year-1-month-old girl was initially admitted to our hospital in March 2023 because of fatigue lasting for more than 4 months and dizziness lasting for approximately 2 weeks. Physical examination revealed an anemic appearance, with pallor of the skin and mucous membranes. No other abnormalities were observed. Her past medical history, personal history, and family history were unremarkable. Laboratory examination after admission revealed severe microcytic hypochromic anemia, with Hb 65 g/L, MCV 61.40 fL, MCH 15.30 pg, MCHC 249 g/L, reticulocyte percentage 2.47%, and absolute reticulocyte count 105 × 10^9^/L. Because the etiology of anemia remained unclear, bone marrow aspiration was performed to evaluate hematopoietic function and exclude hematological disorders. The findings were consistent with microcytic hypochromic anemia. After discharge, the patient received regular oral iron dextran supplementation. Her Hb level temporarily returned to the normal range; however, anemia recurred after discontinuation of treatment. Therefore, she was referred to the Pediatric Gastroenterology Department for evaluation of a possible gastrointestinal cause of recurrent iron-deficiency anemia. Repeat complete blood count showed Hb 114.0 g/L, MCV 71.8 fL, MCH 20.80 pg, MCHC 289.0 g/L, reticulocyte hemoglobin content 25.50 pg, reticulocyte percentage 1.73%, and absolute reticulocyte count 95 × 10^9^/L.Serum gastrin-17 was elevated (>60.00 pmol/L), and the ^13^C urea breath test was negative. To evaluate gastrointestinal function and exclude autoimmune gastritis, serum pepsinogen testing (PGI and PGII) was performed, which showed a pepsinogen II level of 13.60 ng/mL, while other parameters were within the normal range. Gastritis-associated antibody testing was negative. EGD revealed thickened gastric folds with pale mucosa in the gastric fundus and body. Scattered small areas of normal-appearing gastric mucosa were observed between the folds (Fig. 2 A, B). Histopathological examination of gastric biopsy specimens demonstrated moderate superficial chronic inflammation with erosion in the gastric body mucosa, accompanied by fundic gland hyperplasia. Focal layered proliferation of collagen-like fibers was observed beneath the surface foveolar epithelium. Masson’s trichrome staining was positive, consistent with a diagnosis of collagenous gastritis(Fig. 2 C, D). Colonoscopy revealed no significant abnormalities.

After completion of EGD, the patient received hemostatic therapy, gastric mucosal protection therapy, and oral iron supplementation. After discharge, she was treated with omeprazole, aluminum–magnesium carbonate, and L-glutamine sodium oligopeptide granules.At the 2-month follow-up visit, the patient reported no discomfort. Repeat laboratory examination showed normalization of Hb levels. The MCV, MCH, and MCHC values were 73.7 fL, 21.7 pg, and 295.0 g/L, respectively. Although these parameters remained slightly below the normal range, they had improved compared with previous values. Iron metabolism evaluation showed ferritin 4.9 ng/mL, serum iron 6.4 μmol/L, erythropoietin 20.63 mIU/mL, unsaturated iron-binding capacity 73.4 μmol/L, and transferrin saturation 8.02%. At 8 months of follow-up, omeprazole had been discontinued according to the patient’s family’s preference. The patient continued oral iron supplementation and received traditional Chinese medicine treatment according to the family’s preference. Repeat EGD was not performed because the family declined further endoscopic evaluation.

### 2.3. Literature review

A literature search was conducted in PubMed, Wanfang Data, and China National Knowledge Infrastructure (CNKI) databases using the keywords “collagenous gastritis” in English and Chinese. The search was limited to articles published in English or Chinese between December 1989 and December 2025. The exclusion criteria were as follows: cases diagnosed with collagenous colitis, collagenous enteritis, collagenous sprue, or other disorders primarily involving the intestine without definite evidence of gastric collagen deposition; cases diagnosed only with non-collagenous gastric diseases, including common gastritis, chronic gastritis, or lymphocytic gastritis; cases with endoscopic nodular gastric mucosal changes but without histopathological confirmation of CG; duplicate reports; and reports lacking sufficient clinical information, endoscopic findings, or histopathological data for case analysis. A total of 93 patients with CG were identified. Clinical characteristics, endoscopic findings, histopathological features, treatments, and follow-up outcomes were extracted and summarized descriptively.

## 3. Results

Among the 93 reported patients, pediatric cases accounted for approximately 58% (54/93), whereas adult cases accounted for approximately 42% (39/93).

### 3.1. Pediatric patients

Among pediatric patients, the most common clinical manifestations were abdominal pain (57%), anemia (46%), and fatigue (28%) (Table 1). EGD findings varied among different gastric regions, including the gastric body, fundus, and antrum. The gastric body was the most frequently affected region, with nodularity observed in approximately 50% of cases and erythema observed in approximately 26% of cases. Other reported endoscopic findings included mucosal friability, polyps, mucosal atrophy, and erosions. Involvement of the gastric fundus and antrum was less frequently reported (Table 2). Histopathological examination consistently demonstrated marked inflammatory cell infiltration, predominantly involving eosinophils and lymphocytes. Continuous or patchy subepithelial collagen bands were frequently observed beneath the surface epithelium, with collagen thickness exceeding 10 μm in some patients. Masson’s trichrome staining confirmed collagen deposition.

**Table 1:** Clinical manifestations of pediatric patients with collagenous gastritis.

Symptom			
Abdominal pain	31/54	Vomiting	7/54
Anemia	25/54	Diarrhea	7/54
Fatigue	15/54	Hematochezia	4/54
Pallor	14/54	Nausea/Headache	5/54

**Table 2:** Summary of endoscopic features in pediatric patients with collagenous gastritis.

Endoscopic Features									
	Nodularity	Erythema	Friability	Polyps	Nodular elevation	Atrophy	Erosion	Coarseness	Edema
Gastric body	27/54	14/54	8/54	4/54	4/54	4/54	5/54	2/54	3/54
Gastric fundus	19/54	12/54	8/54	1/54	4/54	5/54	4/54	3/54	3/54
Gastric antrum	16/54	9/54	8/54	1/54	1/54	3/54	4/54	2/54	3/54

### 3.2. Adult patients

Among adult patients, the most common clinical manifestations were abdominal pain (41%), abdominal distension (28%), and diarrhea (28%) (Table 3). Unlike pediatric patients, adult CG did not show a preferential distribution among the gastric body, fundus, or antrum. EGD findings across different gastric regions included nodularity (51%), erythema (26%), and inflammatory changes (23%) (Table 4). Histopathological examination revealed dense inflammatory cell infiltration, mainly composed of eosinophils, lymphocytes, and plasma cells. Continuous subepithelial collagen bands were commonly observed, with thickness exceeding 10μm in some cases. Masson’s trichrome staining confirmed collagen deposition. Collagen deposition involving other gastrointestinal sites, including the colon, was reported in some patients.

**Table 3:** Summary of clinical manifestations in adult patients with collagenous gastritis.

Symptoms			
Abdominal pain	16/39	Anemia	7/39
Abdominal distension	11/39	Nausea	4/39
Diarrhea	11/39	Vomiting	4/39
Weight loss	8/39	Dyspepsia	2/39

**Table 4:** Summary of endoscopic features in adult patients with collagenous gastritis.

Endoscopic features			
Nodularity	20/39	Polyps	4/39
Edema	10/39	Depressions	4/39
Gastritis	9/39	Edema	4/39
Mucosal atrophy	6/39	Ulceration	2/39

### 3.3. Comorbidities

In addition to the characteristic clinical features observed in different age groups, several associated conditions were identified. Ten patients (5 pediatric and 5 adult patients) had concurrent Helicobacter pylori infection. Seven patients (5 pediatric and 2 adult patients) showed elevated fecal calprotectin levels. Collagenous colitis was reported in some patients, mainly adults, who presented with symptoms including abdominal distension, diarrhea, and weight loss.

### 3.4. Prognosis

During follow-up, most patients experienced clinical improvement or remained clinically stable, whereas only a small proportion showed disease progression. Among the 54 pediatric patients, one patient developed severe gastric mucosal erosion on endoscopy, one patient showed markedly increased subepithelial collagen deposition on histopathological examination, and one patient experienced symptom recurrence after discontinuation of proton pump inhibitor therapy. The remaining pediatric patients showed improvement compared with baseline.

### 3.5. Review of pathogenesis and clinical characteristics

The pathogenesis of CG remains poorly understood, and several hypotheses have been proposed, including autoimmune mechanisms, epithelial injury, and impaired mucosal repair processes. Current evidence suggests that CG is closely associated with inflammatory responses and immune-mediated mucosal injury.^[1–3]^ Subepithelial collagen deposition may represent an abnormal repair response following inflammation, infection, or toxic injury. One proposed mechanism involves an aberrant immune response to luminal antigens or other stimuli, leading to activation of inflammatory cascades and subsequent excessive collagen accumulation. Therefore, CG may represent a manifestation of a broader gastrointestinal inflammatory disorder rather than an isolated gastric disease entity.^[4,5]^ Previous studies have suggested dysregulated immune responses involving both Th1- and Th2-associated pathways,^[6–10]^ which may contribute to persistent inflammation, eosinophilic infiltration, and fibroblast activation.^[11–15]^ In addition, impaired epithelial repair mechanisms, including altered growth factor signaling, may further promote abnormal subepithelial collagen accumulation.^[16]^ However, whether these immune abnormalities represent primary pathogenic mechanisms or secondary responses remains unclear.

Clinically, CG demonstrates a marked female predominance and a bimodal age distribution, with the first peak occurring during adolescence (10–19 years) and the second occurring predominantly among women older than 60 years.^[17]^ Lagorce-Pages et al classified CG into 2 distinct phenotypes: pediatric and adult forms.^[18]^

Currently, no standardized diagnostic criteria have been established for CG. Diagnosis primarily relies on characteristic histopathological findings combined with supportive endoscopic and clinical features. Histological confirmation remains the diagnostic cornerstone, demonstrating a thickened subepithelial collagen band exceeding 10 μm, usually identified by special staining methods such as Masson’s trichrome staining, together with infiltration of inflammatory cells including lymphocytes, plasma cells, and eosinophils.^[19–21]^ Treatment of CG remains largely empirical and is primarily based on supportive and symptomatic management. Reported therapeutic approaches include acid suppression therapy with H2 receptor antagonists or proton pump inhibitors (PPIs), aluminum-containing mucosal protective agents, iron supplementation for anemia, gluten-free diets in patients with celiac disease-associated CG, corticosteroids, and other immunomodulatory therapies such as mesalamine, sulfasalazine, and parenteral nutritional support. Among these interventions, management of anemia remains particularly challenging. Previous studies have suggested that oral iron supplementation may aggravate gastrointestinal mucosal injury.^[22,23]^ Ferrous sulfate, one of the most commonly used oral iron preparations, has been associated with gastrointestinal adverse effects, particularly in pediatric populations.^[24,25]^ For pediatric patients with CG, a gluten-free diet may be considered as an initial therapeutic approach, particularly in cases associated with celiac disease.^[26]^ Corticosteroids may serve as bridging therapy, leading to clinical remission and improvement of anemia.^[27]^ Vedolizumab, an anti-α4β7 integrin monoclonal antibody, may represent a potential treatment option for refractory CG associated with collagenous colitis, although further clinical studies are required to confirm its efficacy.^[28]^

## 4. Discussion

Collagenous gastritis (CG) is a rare gastric disorder characterized by thickening of the subepithelial collagen band and chronic inflammatory cell infiltration. Pediatric CG differs significantly from adult CG in clinical presentation and disease distribution. Pediatric patients mainly present with upper gastrointestinal symptoms, particularly abdominal pain and iron-deficiency anemia, whereas adult patients are more likely to exhibit diffuse gastrointestinal involvement, including collagenous colitis and chronic watery diarrhea. Endoscopically, pediatric CG typically presents with diffuse erythema, edema, and nodular elevations of the gastric mucosa. The mucosa may appear irregular with a nodular or scar-like appearance and may be accompanied by erosions or ulcers. Among these findings, nodularity is considered one of the most characteristic and common endoscopic features. The differential diagnosis of CG includes several gastric disorders with overlapping endoscopic appearances. Ménétrier disease is characterized endoscopically by markedly enlarged and edematous gastric folds, particularly in the gastric body and fundus, with a cerebral convolution-like or nodular appearance. Histologically, it demonstrates foveolar epithelial hyperplasia, glandular abnormalities, and increased mucus cell proliferation, which are essential for diagnosis.^[29]^ Helicobacter pylori-associated gastritis commonly presents with erythematous and granular nodular changes in the gastric antrum, sometimes accompanied by superficial erosions.^[30]^ Diagnosis primarily relies on histopathological examination. In children with celiac disease involving the stomach, endoscopic findings are usually normal, although some patients may demonstrate antral erythema, mild edema, or small nodular changes. Histologically, increased intraepithelial lymphocytes are typically observed.It should be noted that CG may cause secondary glandular atrophy, leading to potential misdiagnosis as atrophic gastritis.^[31]^ Therefore, definitive diagnosis requires histopathological evaluation of gastric biopsy specimens. In Case 1, the patient had undergone gastroscopy at a local hospital; however, insufficient awareness of CG may have contributed to delayed recognition and the absence of timely gastric biopsy evaluation. Therefore, CG should be considered in children with unexplained anemia, with or without abdominal pain. In Case 2, anemia was the predominant manifestation. Bone marrow aspiration was performed to evaluate hematopoietic function and exclude hematological disorders, which represented an important step in the differential diagnosis.

In the present study, both pediatric patients initially presented with pallor or anemia, and their endoscopic and histopathological findings were consistent with CG. Treatment was based on supportive management, focusing on gastric mucosal protection and correction of anemia, with favorable clinical outcomes. L-glutamine sodium oligopeptide granules contain glutamine and sodium gualenate. Glutamine is an important energy substrate for gastrointestinal epithelial cells and may promote mucosal repair by enhancing DNA synthesis and protein synthesis.^[32]^ Aluminum–magnesium carbonate is a gastric mucosal protective agent with a layered molecular structure. After administration, it can form a protective coating on the gastric mucosal surface and potentially enhance mucosal defense.^[33]^ However, the efficacy of these agents in pediatric CG has not been specifically demonstrated in clinical studies. In our patients, both medications were administered because endoscopic examination revealed fragile, bleeding-prone gastric mucosa accompanied by varying degrees of inflammation. The subsequent clinical improvement suggests potential symptomatic benefits; however, further studies are required to determine their therapeutic efficacy in CG. Long-term management of pediatric CG requires monitoring of anemia severity, gastric mucosal recovery, and potential steroid dependence. Follow-up assessments including Hb levels, fecal occult blood testing, fecal calprotectin measurement, and repeat endoscopic evaluation when clinically indicated may help evaluate mucosal healing and changes in subepithelial collagen deposition.

In summary, pediatric CG is a rare condition that commonly presents with abdominal pain and anemia. CG should be considered in children with recurrent unexplained anemia, particularly when accompanied by abdominal discomfort. Early endoscopic examination combined with histopathological evaluation and special staining is essential for accurate diagnosis.

## Acknowledgments

The authors sincerely thank all investigators and patients who contributed valuable information and support to this study.

## Author contributions

**Writing—original draft:** Baoqi Chen, Haiyan Liu, Jinming Gong, Yuan Chen, Wenxin Shi, Lu Guo, Lifeng Sun.
